# Convolutional Neural Network Based Approach to In Silico Non-Anticipating Prediction of Antigenic Distance for Influenza Virus

**DOI:** 10.3390/v12091019

**Published:** 2020-09-12

**Authors:** Majid Forghani, Michael Khachay

**Affiliations:** Krasovsky Institute of Mathematics and Mechanics, 620990 Ekaterinburg, Russia

**Keywords:** influenza, antigenic distance, vaccine, convolutional neural network, evolution

## Abstract

Evaluation of the antigenic similarity degree between the strains of the influenza virus is highly important for vaccine production. The conventional method used to measure such a degree is related to performing the immunological assays of hemagglutinin inhibition. Namely, the antigenic distance between two strains is calculated on the basis of HI assays. Usually, such distances are visualized by using some kind of antigenic cartography method. The known drawback of the HI assay is that it is rather time-consuming and expensive. In this paper, we propose a novel approach for antigenic distance approximation based on deep learning in the feature spaces induced by hemagglutinin protein sequences and Convolutional Neural Networks (CNNs). To apply a CNN to compare the protein sequences, we utilize the encoding based on the physical and chemical characteristics of amino acids. By varying (hyper)parameters of the CNN architecture design, we find the most robust network. Further, we provide insight into the relationship between approximated antigenic distance and antigenicity by evaluating the network on the HI assay database for the H1N1 subtype. The results indicate that the best-trained network gives a high-precision approximation for the ground-truth antigenic distances, and can be used as a good exploratory tool in practical tasks.

## 1. Introduction

The Influenza virus has a high morbidity and mortality rate, leading to about 3–5 million cases of severe illnesses, up to half a million deaths around the world annually, and accordingly, high economic losses [1]. The World Health Organization (WHO) continuously monitors the viral acts, including both epidemic and pandemic, and decides the strategies to fight with the virus. Currently, the vaccination appears to be the most useful option in the struggle against the influenza virus. Despite its efficiency, this method requires permanent reviewing and updating due to continuous viral evolution [2]. Hence, the prediction of viral genetic and antigenic evolution has a great impact on the applicability of the vaccine components recommended by the WHO.

Typically, the influenza virus has two main surface antigens, hemagglutinin (HA) and neuraminidase (NA) proteins, which are vital for replication of the virus. There are four main types of the influenza virus, A, B, C, and D, among which A and B lead to serious public health issues. Type A is further divided into subtypes based on 18 different HA (H1-H18) and 11 different NA (N1-N11) so that, theoretically, it is 198 possible different combinations of these proteins, which enables the virus to infect a wide range of different hosts [3]. Currently, the HA protein of subtypes H1N1, H3N2, and B are considered for vaccine composition, from which H1N1 has been chosen for the current study.

The H1N1 subtype is a rapidly evolving virus, whose nucleotide mutation rate is estimated at about 0.76 [4] for a genome per replication. The alternations of the surface proteins, such as HA and NA, result in the reduction of virus recognition by the host’s immune response and a novel antigenic variant emerges. Ultimately, antigenic variants can be generated by two well-known processes. The former one called *antigenic drift* is based on accumulated mutations, and typically leads to a seasonal epidemic. The latter process referred to as *antigenic shift* is achieved from the re-assortment of two different subtypes of the virus, and may cause a pandemic [5]. Therefore, the prediction of changes in antigens and their direction over time becomes a topic of numerous research papers in the field of viral evolution modeling (see, for example, [6,7]).

Basically, the primary target of an immune response against the influenza virus is considered its HA antigen. The representativeness of the HA antigen on the virus surface is approximately four times greater than NA [8]. HA is also the main component of the influenza vaccine [9], and great effort is directed towards studying this antigen and its mutations. Therefore, in this paper, the HA sequence is considered as raw input for the prediction of antigenic variants.

The effectiveness of a vaccine directly depends on antigenically matching its composition strains with current dominant circulating viruses. Sometimes, the substitution of even a few amino acids can dramatically change the antigenic characterization of the virus. For instance, Smith et al. [10] showed that a single substitution is responsible for antigenic alteration between two antigenic clusters, BE89 and SI87. That is why the majority of antigenic modeling approaches, along with raw HA sequences, rely on additional data, such as information about protein structure [6] and the amino acid neighbor effect [11,12]. In this paper, we use the physicochemical properties of amino acid to encode raw input sequences and produce a multi-aspect representation of the studied virus strains.

The main challenge in vaccine development is that it is carried out under time pressure. The efficiency of a vaccine depends on the rapid quantification of their antigenic relatedness and detection of antigenic variants [5]. To evaluate the antigenic similarity between different strains of the influenza virus, the classic Hemagglutination-Inhibition (HI) assay laboratory procedure is performed (see, for example, [13]). The HI assay mainly evaluates how antibodies against a reference strain can efficiently bind and block an antigen of another (test) strain of the virus. The high value of the HI *titer* indicates a high degree of antibody binding [14].

Although the HI assay is a gold standard and widespread technique used in vaccine production, it suffers from the following shortcomings: (i) It is rather time-consuming and expensive; (ii) the obtained assays are generally unsuitable for quantitative analyses and difficult in interpretation [10]; (iii) the obtained measurements are unstable and noisy; (iv) antigenic pairwise comparison between some strains can be missed in the HI table [5]; and finally, (v) the results can be influenced by the effects of so-called *egg adaptation* and NA activity [15].

Therefore, in recent decades, computer-aided approaches have been developed to quantify and predict antigenic evolution [6], which are mainly based on the *antigenic distance* concept. There are two widely used definitions of antigenic distance [16,17], driven from the HI table. If the $H_{i,j}$ represents the obtained HI titer of antibody based on the antiserum of strain *j* to inhibit the antigen of strain *i*, then the antigenic distance can be defined as follows:

(1) $$d_{1}\left(i,j\right)=\log_{2}\left(\frac{H_{j,j}}{H_{i,j}}\right)$$

(2) $$d_{2}\left(i,j\right)=\sqrt{\frac{H_{i,i}\times H_{j,j}}{H_{i,j}\times H_{j,i}}}.$$

In this paper, we rely on definition (1), since Equation (2) requires a homologous titer of both strains *i* and *j*, while there exists a possibility that one of them can be missed in the dataset. $d_{1}$ is very close to the concept of a standardized $\log_{2}$ titer [18].

In $d_{1}$, the value $H_{j,j}$ is replaced by a maximum titer observed for antiserum *j* against any antigen in the HI table if there is a titer $H_{i,j}$, such that $H_{i,j}>H_{j,j}$ [19]. A drawback of the HI assay technique is that it is carried out on the specific amount of strains, while a sequence-based antigenic distance can be employed on all strains collected and sequenced in any period of time. An instance of such a distance, called $P_{epitope}$, has been introduced by Gupta et al. [20].

Various sophisticated computational approaches have been developed in order to predict the predominant strains of the upcoming season, as well as the vaccine strains which basically require determination of the antigenic similarity between influenza strains. An overview of recent computational approaches is presented in [6], in which the approaches are categorized into four main categories: phylogenetics and population genetics; statistical methods; epidemiological models; and other methods, which are based on graph or information theories. The prediction approaches may utilize different information types, such as the viral sequence, HI assay data, protein structure, and epidemiological information. With the progress in computational technology and its accessibility in the recent decade, the implementation of more sophisticated models has become available through parallel computing frameworks.

In this paper, we propose a novel approach to approximate antigenic distances based on deep Convolutional Neural Networks (CNNs). Deep convolutional architectures date back to the seminal paper [21], inspired by the visual cortex [22,23], which significantly extended the conventional theories of ensemble learning (see, for example, [24,25]) and polyhedral separability [26], and became a successful algorithmic framework in classification, pattern recognition, regression, and natural language processing. The convolutional neural network utilizes a hierarchical architecture, which enables modeling of complex functions through assigning the suitable output to input, performing multilevel automatic feature extraction. The hierarchical structure allows us to extract low features at early levels of the network and combine them in further layers to form high-level features. The transformation of more abstract low-level features into high-level ones is carried out by convolutional layers. A prominent advantage of CNN is that it does not require feature engineering and the features are automatically selected during the training process.

### 1.1. Convolutional Neural Networks

Through multi-processing layers, CNN can construct multiple levels of abstraction. In addition to the conventional *fully connected layers*, a typical CNN consists of a number of more advanced layers, where among them are *convolutional*, *rectified linear units* (ReLU), *pooling*, *dropouts*, and their combinations [27]. Both convolutional and pooling layers define the feature extraction process. While convolutional layers (in combination with ReLU) extract the features, pooling layers compress the convolutional layer output to reduce the dimension of the feature space. Further, the extracted features are fed into a fully connected layer which serves as a classifier to assign the suitable output into the input object. The performance of CNN depends on two critical factors: its architecture and hyperparameter tuning. Generally speaking, there is no direct rule in the selection of suitable architecture, and it is often chosen empirically. Moreover, the hyperparameter tuning is performed through computational experiments on the network. Like other modeling and classification methods, CNN suffers from overfitting that is related to the mentioned factors, as well as the data characteristics. It happens when CNN is not able to capture the regular pattern from data. However, despite these difficulties, CNN applications are rapidly growing and indicate high performance in pattern recognition and classification tasks.

### 1.2. Related Work

In this section, we give a short overview of a number of published results on the prediction of the antigenic distance between influenza virus strains.

First, we noticed that Equations (1) and (2) are not the only possible way to define a concept of the antigenic distance. For instance, the authors of [20,28,29] proposed various alternative definitions for this concept, based on several statistics describing point mutations in the HA sequence. The most recent results in this field were reported by Skarlupka et al. in [30].

Du et al. [31] introduced a 12-dimensional feature space for comparing pairs of virus strains. Their first five features describe variations in given antigenic sites (epitopes), the next group of five features presents some target physicochemical properties of the amino acids along the sequence, and the final two are related to receptor-binding and glycosylation.

By learning at the class of Naïve Bayes classifiers in the constructed feature space, the authors managed to accurately identify the antigenic cluster of H3N2 and H1N1 subtypes [31,32].

Yao et al. [33] proposed a joint random forest regression algorithm predicting the antigenicity of the influenza virus on the genetic information encoded in terms of amino acid substitution matrices. Applying this approach, the authors managed to reproduce the results for antigenic cartography of the H3N2 subtype obtained initially by Smith et al. in their seminal paper [10].

Wang et al. [34] introduced an imputation-based approach for recovering missed values in the HI assay table and drawing the appropriate antigenic cartography. The robustness of the proposed method was justified numerically by 10-fold cross validation.

Cui et al. [35] utilized entropy of a specific site, as well as the relationship between mutation occurrence in that site and antigenic variation to recognize the critical position in the sequence. They clustered the well-known *AAindex* database [36], taking into account the mutual information between physicochemical changes at a critical position and antigenic relationship. To construct a feature space, they used a representative from each cluster to encode the amino acid mutation located in the critical positions with respect to query and reference strains. Further, they employed multiple stepwise regression to predict the antigenic variants.

To estimate the antigenic distance, Suzuki [37] relies on specific physicochemical properties and structural information, such as the distance between $C_{\alpha }$ atoms of the specific position and receptor-binding. The author evaluated the effectiveness of a vaccine strain for a season by computing the average antigenic distance between the query strain and circulating viruses in the season.

Neher et al. [18] proposed two models based on the HA sequence for predicting the antigenic evolution of the influenza virus. The first model employs the length of the path between a test and reference viruses in the phylogenetic tree as a parameter to explain the virus closeness, while the second model relies on the contribution of amino acid substitutions associated with antigenicity. Evaluating both models on four influenza virus lineages achieve high accuracy of prediction.

In the recent decade, deep learning became increasingly important in the various bioinformatics domains, especially in *omics research* [38]. Among them are protein structure prediction [39], gene expression regulation [40,41,42], predicting the sequence specificities [43], and protein classification [44]. Recently, deep learning has been applied to predict the mutation of the influenza virus [45], pathogenicity classification of H5 avian influenza [46], as well as time-series modeling for the recently emerging COVID-19 outbreak [47]. An inevitable problem in omics research is the representation of raw biological sequences, that is, amino acid sequence, as a network input. This issue can be tackled by encoding the raw sequence, which is usually carried out using one-hot [44], amino acid property encodings [48], or embedding methods [45].

Although the aforementioned approaches appear to be promising and achieve sufficient accuracy, all of them, to the best of our knowledge, suffer from a number of shortcomings, as follows:

(i) They deal with a point mutation as a single event, while it is widely known that amino acids located at some specific position affects its close, and even not so close (due to the folding) neighbors in the protein sequence.
(ii) Despite the wide engagement of the deep learning principle in biological research, all the known models of the antigenic evolution rely on manual feature engineering.
(iii) Previous research did explicitly take into account the *temporal factor*, that is, the date/time when a certain virus strain was isolated for the first time. Therefore, all of them were not non-anticipating, since they relied on measurements describing future substitutions.

In our research, we try to bridge this gap by proposing a novel non-anticipating approach for prediction of antigenic distances based on Convolutional Neural Networks.

### 1.3. Our Contribution

The contribution of this paper is three-fold:

(i) We propose a novel approach for prediction of the antigenic distance based on convolutional neural networks trained in a few-dimensional physicochemical feature space of amino acids, constituting HA sequences of the compared strains of the influenza virus.
(ii) By employing the Grid-Search method for tuning the hyper-parameters of a neural network, we choose the best CNN architecture, and the performance of the obtained model exceeds the well-known SqueezeNet CNN model [49] taken as a baseline both by the performance and number of learnable parameters.
(iii) In addition, relying on experiment scenarios proposed in [18], we evaluate the performance of our best CNN model and show that it provides quite an acceptable prediction quality. All the source code, auxiliary scripts, trained networks, and figures are freely available at https://github.com/ForghaniM/FLU.

The remaining part of the paper is organized as follows. In Section 2, we explain the initial data collection, methodology, and design of our experiments. Thus, Section 2.1 provides a description of the source and characteristics of the initial HI data. In Section 2.2, we explain our approach to encoding the amino acid sequences. Then, Section 2.3 and Section 2.4 introduce architectures of the convolutional neural networks employed in subsequent experiments. Then, in Section 2.5 entitled "Experimental Design", we provide a general scheme of our experiments. Further, in Section 3, we report our experimental results, including performance comparison of the considered models, their prediction accuracy, and the number of trainable parameters influencing the total time consumption. Then, in Section 4, we discuss the properties of the considered models and some biological aspects of the obtained results in more detail. Finally, in Section 5 we summarize our results and give a short overview of future work directions.

## 2. Materials and Methods

**Figure 1:**
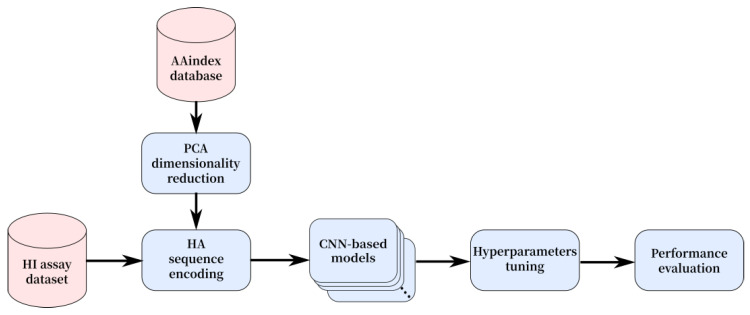
General diagram of the proposed research.

In this section, we describe the proposed approach for the prediction of antigenic distance for the influenza virus of the H1N1 subtype. We start with data collection, preprocessing, and encoding of the genomic sequence; then we introduce the used CNN architectures, design of the subsequent experiments, and general scenarios of hyperparameter tuning for high-accuracy prediction of the antigenic distance (see Figure 1).

### 2.1. Data Collection

#### 2.1.1. HI Assay Dataset

**Figure 2:**
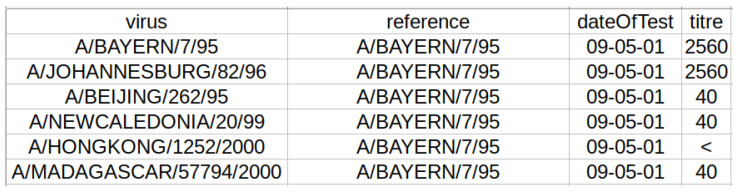
Each HI assay entry includes identifiers of the test and reference viruses, date of experiment, and the measured titer.

We took HI assay data from the repository [50] located at the University of Glasgow. This repository provides a dataset of 48,707 entries related to the H1N1 subtype, obtained from 1989 to 2010 as a result of the HI assay for 4436 test viruses against 92 reference strains. Each entry of this dataset is composed of two strain identifiers (test and reference), the date of the laboratory experiment, and the obtained HI titer (see, for example, Figure 2). Unfortunately, the HA sequence is available only for 506 out of 4436+92 test and reference strains. This information can be extracted from the well-known GISAID EpiFlu database [51] by the GISAID identifiers provided in the entries of the initial dataset.

Therefore, out of 48,707 initial HI assay entries, we filtered out 28,028 due to the lack of HA sequence information and used the remaining entries for the subsequent study. Hereinafter, we use HI assay data for computing (by Formula (1)) the ground-truth values of antigenic distances for our prediction models.

#### 2.1.2. Hemagglutinin Sequence

The hemagglutinin (HA) sequence, also referred to as HA0, consists of two parts: the globular head domain, that is, HA1, and stalk domain, or HA2. Typically, an HA0 sequence consists of 565 amino acid positions, where the 18th to 343rd positions belong to HA1, whilst the remaining subsequence belongs to HA2. We aligned all 506 available HA sequences by using the MUSCLE package from the well-known MEGA X software version 10.1 [52] in order to determine the longest common subsequence (fragment) of HA protein. In our case, this common fragment of the HA sequence has a length of 304 amino acids (including one gap) and is located between the 18th and 320th positions, according to the HA0 domain. Thus, the protein sequence of test and reference viruses belong to the HA1 domain, and include the antigenic and receptor-binding sites (see Table 1).

### 2.2. Amino Acid Sequence Encoding

In modeling of antigenic variants (see, for example, [31,35]), encoding techniques have a direct impact on the predicted results, since they determine how the mutations are represented in the model. To reflect amino acid substitutions in a more descriptive way, we applied physicochemical properties to measure up a quantitative value of the observed mutations. The rich collection of amino acid properties is located in the AAindex database. AAindex consists of three data types. The first of them, referred to as AAindex1, is a collection of indices representing the amino acid properties, such as hydrophobicity, residue volume, and molecular weight. The second collection, AAindex2, consists of mutation matrices. Finally, AAindex3 includes the set of protein pairwise contact potentials matrices.

**Figure 3:**
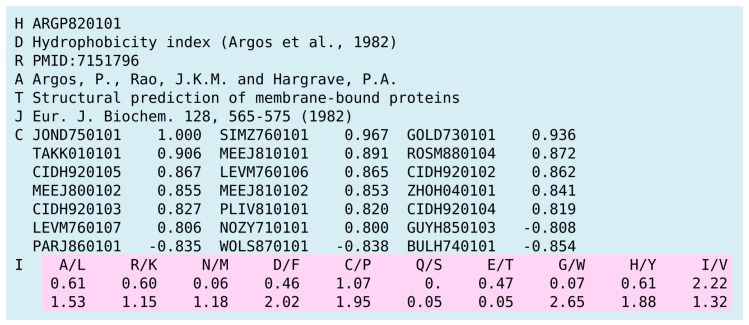
An example of the AAindex1 entry representing the hydrophobicity index. The values assigned to amino acids are highlighted in pink.

For the encoding task, we used the AAindex1 collection only. Each entry, also referred to as the *amino acid index*, is a vector of 20 real numbers, representing a physical or chemical property of 20 standard amino acids (see Figure 3 for details).

**Figure 4:**
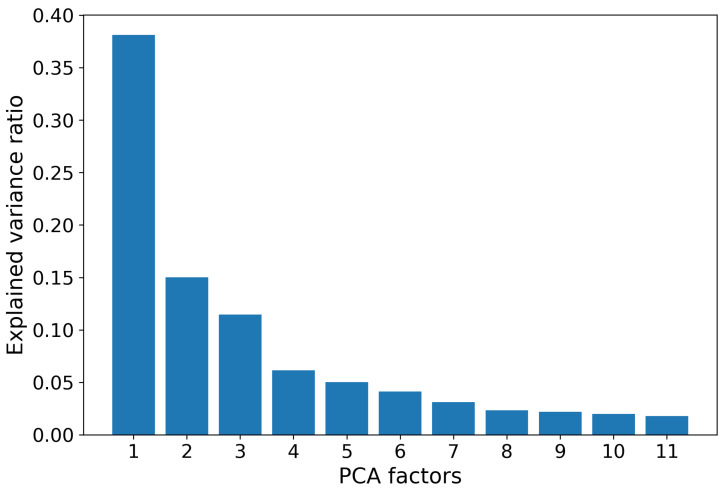
Variance ratios explained by the first 11 factors obtained with application the PCA to AAindex1 database. Total explained variance is about 91%.

After filtering out the indices with missing values, the remaining 553 entries were normalized for further computations. To address possible high dependence between the obtained 553 indices, we applied the standard procedure of the Principle Component Analysis (PCA). The first 11 factors, which we call *synthetic indices*, explain about 91% of the total variance, while the contribution of each subsequent factor is vanishing. Therefore, in the sequel, we restrict ourselves by these first 11 indices (see Figure 4). Further, we normalize the obtained synthetic indexes to fit witin the range [0,255].

### 2.3. Input Tensor Structure

**Figure 5:**
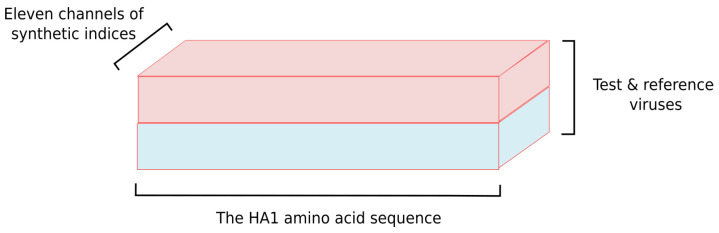
The network input tensor represents the HA1 amino acid sequence of test and reference viruses encoded by 11 synthetic indices from AAindex1.

Our approach to predicting antigenic distances between strains of the influenza virus is based on deep convolutional neural networks trained on the aforementioned HI table. As usual, an input data structure for such networks is represented in the form of multidimensional numerical arrays or *tensors*. The synthetic indices provide 11 different representations of amino acid sequences, which will be located in different channels of the network input tensor. Since an arbitrary entry of the HI table represents a measurement, which relates to a pair of test and reference viruses, we specify input for our neural networks as a tensor of shape 304×2×11. Here, 304 stands for the length of the HA sequence, and 2 stands for a pair of strains compared on 11 distinct synthetic indices (see Figure 5 for details).

### 2.4. Architectures of the Examined Networks

Convolutional Neural Networks (CNN) is a state-of-the-art class of deep learning algorithms providing the multilevel representation of input data, which is usually applied to learn in classification or regression estimation. As is known (see, for example, [27]), Convolutional Neural Networks give extremely high-performance results in image analysis, including such hard-to-solve tasks as object detection [54] and semantic segmentation [55,56]. Another prominent advantage of CNNs is the reduced need for a preliminary construction of the feature space. In conventional machine learning, such a procedure is also referred to as *feature engineering* (FE) [57], and as a rule, is performed by a researcher manually. As is also known, FE is hard to formalize, is time-consuming, and is a complicated procedure, crucially depending on characteristics of the initial data and having a high impact on the performance of the resulting model. Any time when the initial dataset is updated, especially in the case of evolution modeling when a new mutation has a high impact on the objective function, the FE should be replayed as well.

**Figure 6:**
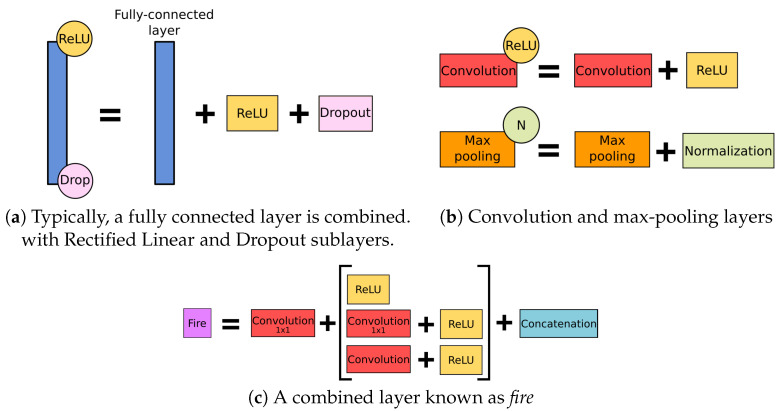
The layers used in the examined networks.

Typical CNN architecture consists of two main components—namely, a *feature learning* module intended to extract the most relevant features from the initial dataset and solve the feature engineering task automatically, and a *classification or regression estimation* module representing the desired model. In turn, even a simple feature learning module consists of several *layers*, including the *convolutional layer*, *activation layer*, *pooling*, a *dropout*, and their combinations (Figure 6).

**Figure 7:**
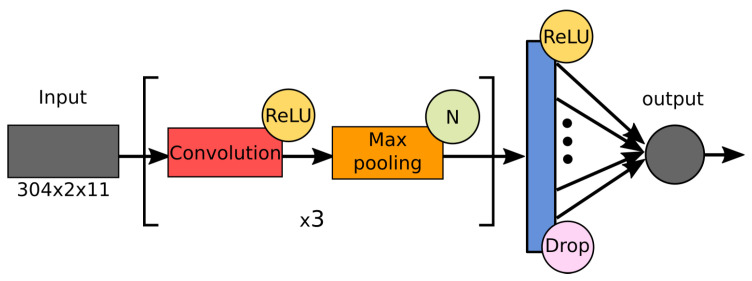
Common architecture of the tested networks, M1-M32.

In our study, we were faced with a noisy HI assay dataset containing a number of partially duplicated records that contradicted each other. To ensure highly reliable predictions, we deliberately focused on the most compact CNN architectures that minimized the number of learnable parameters while simultaneously providing high prediction accuracy levels. Therefore, our main architecture straightforwardly follows the well-known AlexNet [58]. In our experiments, we carried out a numerical comparison for a number of networks, which we called M1–M32; each of them has quite similar architecture (Figure 7) and differs from each other by some values of *hyperparameters*, including the number of convolution levels and properties of used convolution kernels (see Table 2 for details).

**Figure 8:**
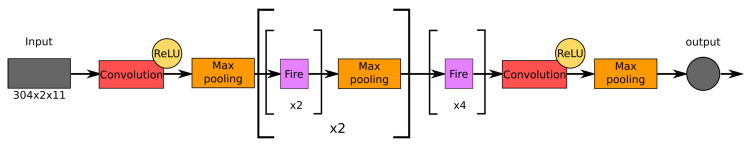
Architecture of SqueezeNet used as a baseline network.

As a baseline in our experiments, we employed the SqueezeNet (Figure 8), which is known for having the smallest modern CNN [49] providing a high accuracy level on the Top Five ImageNet competition [59].

We should notice that both the initial AlexNet and SqueezeNet are classification networks. To adapt their architectures to our task of antigenic distance prediction, we replaced their final layers with the single output inner product layer and used Mean Absolute Error (MAE):

(3) $$MAE=\frac{\sum_{i=1}^{n}\left|y_{i}^{Real}-y_{i}^{Pred}\right|}{n}$$

as a learning criterion, where $y_{i}^{Real}$ and $y_{i}^{Pred}$ are the ground truth and predicted antigenic distances, respectively, and *n* is the size of a dataset.

### 2.5. Experimental Design

We partitioned our experiments into two groups. For the former, we called it *temporal*, and for each virus strain, we took into account the explicit information about the date when this strain was isolated for the first time. Hence, we consider the initial HI assay dataset in the context of the time series. For the latter one, which we called *static*, we rely on the experiment scenario proposed in [18], where all the measurements from the HI assay dataset are considered simultaneously, without any explicit dependence on the isolation date of the related strains.

#### 2.5.1. Temporal Experiments

As mentioned above, the considered HI assay dataset includes measurements dated from 1989 to 2010. Due to the high sparsity of the dataset for the years 1989 to 2000, we trained all the models for prediction over the period 2001–2009. This group consists of four closely related experiments, where in each of them, all the networks were trained to predict antigenic distances for a certain target year with respect to the prior information (prehistory). Model validation was carried out for the entries of the HI assay dataset, where the test virus was isolated exactly in the target prediction year. To perform a fair performance comparison in each experiment, all the networks were trained (tested) under identical conditions. In the first experiment $T_{all}$, we employed all the available prior information up to the target year, while in the other three experiments, $T_{3}$, $T_{4}$, and $T_{5}$, the prehistory was restricted to three, four, and five years immediately preceding the target year, respectively. For each experiment, we found the top five models with respect to mean MAE in the time period 2001–2009 using the Grid-Search algorithm in the space of hyperparameters specified in Table 2. For reference, the SqueezeNet [49], which we used as a baseline model, consists of 18 convolutional layers and has about 886,000 trainable parameters. The obtained results are presented in Section 3.1.

#### 2.5.2. Static Experiments

In this group of the experiments, we relied on two scenarios, the *Titer* and *Virus*, proposed by Neher et al. in [18] for the comparison of their Tree and Substitution prediction models of the standardized HI titers. As the authors of the cited work, in both experiments, we restricted ourselves to a seven-year prediction period (from 2003 to 2009, in our case).

According to the former scenario, we carried out 10-fold cross-validation within the entire dataset. On the other hand, for the latter one, we fetched all the distinct virus strains, where after that we performed 10-fold validation each time, excluding from the training all the measurements concerning the viruses that belonged to the validation part.

In both experiments, for the evaluation, we took the best models (among M1–M32), comparing their performance with the baseline SqueezeNet CNN. The obtained results are reported in Section 3.2.

## 3. Results

We implemented all experiments in Caffe [60], one of the most well-known frameworks for deep learning. For training and validation of all the models (networks), we used a computer with two 8-core Intel® Xeon® E5-2650 (2.6 GHz), 64 GB RAM, and 1 GPU Tesla K40m (with 12 GB GDDR5).

### 3.1. Temporal Experiments

**Figure 9:**
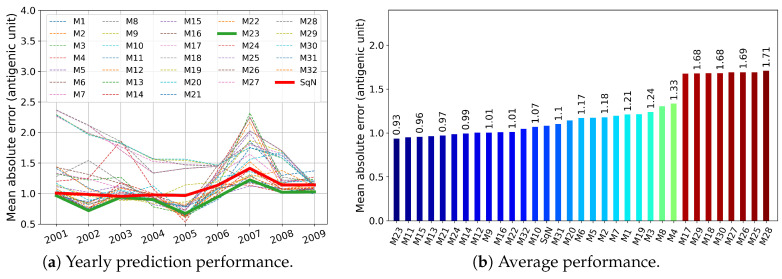
Experiment $T_{all}$, where all models were trained on the unrestricted prehistory.

**Figure 10:**
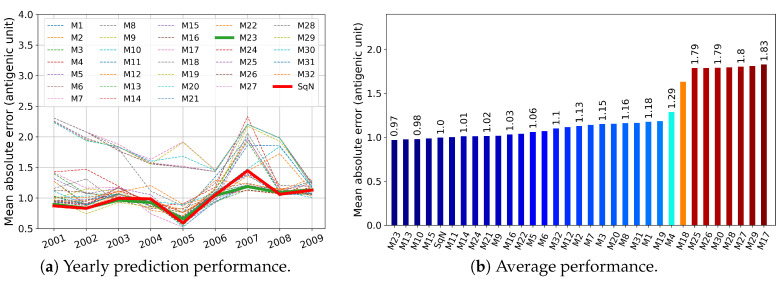
Experiment $T_{5}$: the models were trained on a five-year prehistory.

**Figure 11:**
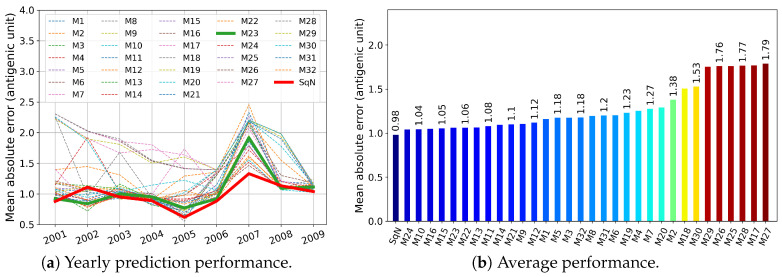
Experiment $T_{4}$: the models were trained on a four-year prehistory.

**Figure 12:**
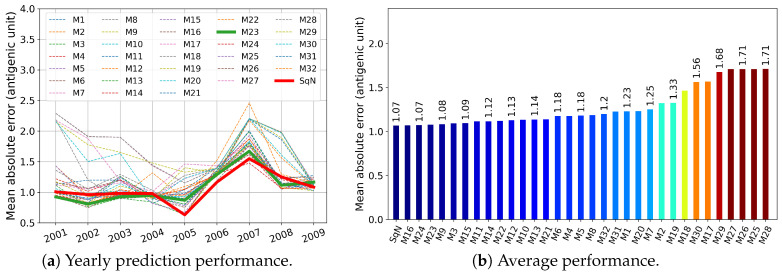
Experiment $T_{3}$: the models were trained on a three-year prehistory.

In this section, we report the results obtained in experiments $T_{all}$, $T_{3}$, $T_{4}$, and $T_{5}$ described in Section 2.5.1. First, in Figure 9, Figure 10, Figure 11 and Figure 12, we present these results graphically. In particular, each of the Figure 9a–Figure 12a, for each examined network and each target year from 2001 to 2009, displays the obtained Mean Absolute Error (MAE) calculated by Formula (3). At each figure, we highlight the plots related to the baseline SqueezeNet (SqN) and the model M23 belonging to the most part of the Top Five short-lists presented in Table 3.

Then, in Table 3, for each experiment $T_{5}$, $T_{4}$, and $T_{3}$, we report annual mean absolute errors for the best five models. The only exception we made was for $T_{all}$, where we included the data concerning the baseline model SqueezeNet, which did not belong to the list of Top Five performers.

As can be seen from Table 3, for a trade-off in MAE <4%, and <5%, we could reduce the prehistory to only 5 or even 4 years, still preserving the MAE of the best model by less than 1 antigenic unit. Moreover, the best five models in both experiments $T_{all}$ and $T_{5}$ obtained MAE less than one antigenic unit.

As can be seen in Figure 9 and Figure 10, among all network variants, M23 outperforms the others—even the baseline model—and achieves minimum MAE of about 0.93 antigenic units, while due to the experimental design, the network does not receive any prior information during the training procedure about the test virus isolated in the target year.

On the other hand, for experiments $T_{all}$ and $T_{5}$, there exist models (M11 and M10, respectively) that have less complexity in structure. For example, taking into account the results of the experiment $T_{all}$, surprisingly, the M11 model wins the second outperformed position, while compared to M23 and SqueezeNet, it requires network parameters about eight times less (see Table 2).

### 3.2. Static Experiments

In this section, to proceed with further investigation of the two models M23 and SqueezeNet selected in the experiments $T_{all}$, …,$T_{3}$, we carried out the experiments *Titer* and *Virus* described in Section 2.5.2 following the scenarios proposed in [18]. Actually, we performed two performance evaluations for the selected models by using the well-known 10-fold cross-validation technique.

**Figure 13:**
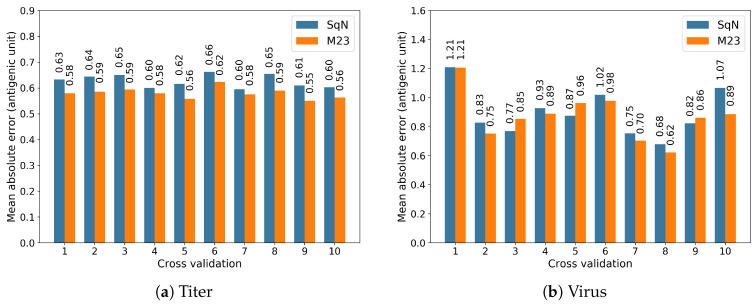
The results of 10-fold cross-validation for models M23 and SqueezeNet.

The results obtained at each iteration of the cross-validation are reported in Figure 13a,b, respectively.

Further, in Table 4, we summarize the obtained performance comparison results in terms of the accumulated Mean Absolute Errors.

**Figure 14:**
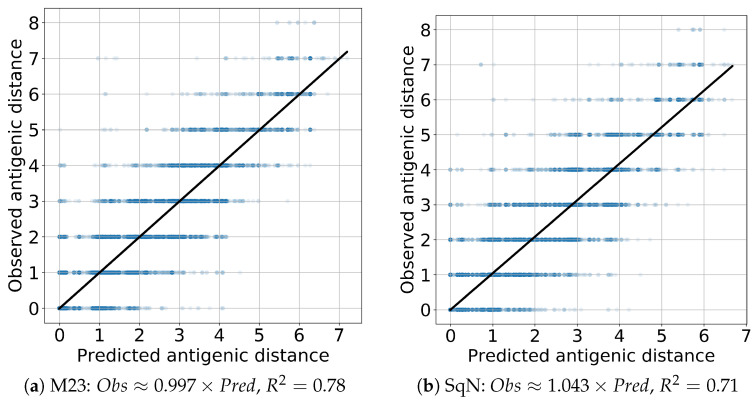
Titer: linear regression.

**Figure 15:**
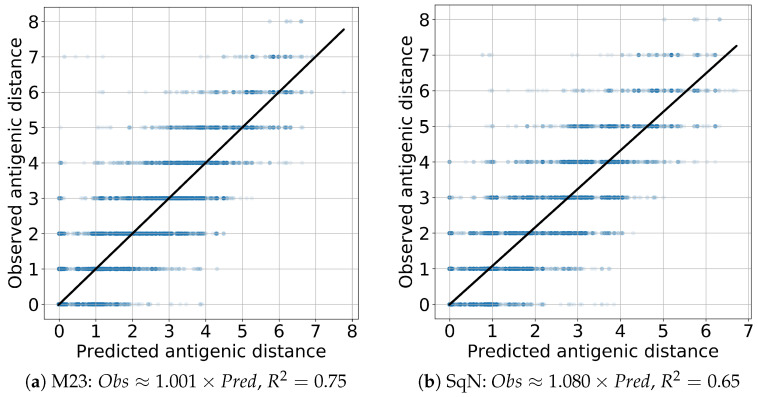
Virus: linear regression.

Finally, to evaluate the prediction performance of both models M23 and SqueezeNet, we estimated the linear regression Predicted∼Observed and $R^{2}$ scores. In all cases, the coefficient of the independent variable appears to be close to the true value 1 with the absolute error being, at most, 0.08. The results obtained are presented in Figure 14 and Figure 15 for the *Titer* and *Virus* experiments, respectively.

#### Comparison with Previous Results

As we mentioned in Section 2.5.2, the reported results were obtained following the experiment scenarios *Titer* and *Virus* proposed in paper [18] for the comparison of two standardized HI titer prediction models entitied by Phylogenetic Tree and Substitution. Our final prediction results presented in Table 4 and Figure 14 and Figure 15 appear to be quite comparible with those in (Table 1 and Figure 2 in [18]), although their direct comparison is complicated by the difference in initial datasets. On the other hand, accuracy of the obtained results outperforms the results reported by Bedford et al. in (Table 1, Col. A/H1N1 in [61]).

## 4. Discussion

In this study, we have demonstrated that the model based on convolutional neural network is able to predict the antigenic distance between strains of influenza virus using their HA1 sequence. There are known various pieces of research aiming at predicting the antigenic distance or variants for the influenza virus [6]. However, our approach differs from predecessors in several ways:

- All the models proposed in this paper are fully *non-anticipating*, that is, they were trained to predict antigenic distances for a given year without taking into account any information concerning future events, such as high-impact substitutions or test virus relationships in a phylogenetic tree. Therefore, all predictions were carried out on the basis of the prehistory exclusively.
- Unless something was a major part of conventional research [18,62], tackling the protein sequences as alphabetic strings, we used a number of *physicochemical properties* of the constituent amino acids presented in the AAindex1 dataset to encode the HA protein sequence that provides a multi-representation of input genetic data and specifies mutation patterns in a more descriptive way.
- Unlike most papers which adopted manual feature engineering [35,62], for example, those based on prior knowledge about antigenic sites and receptor-binding, our approach relies on the advantage of the convolutional neural network framework to provide fully *automatic feature extraction* by automatically assigning the most relevant features for prediction of the antigenic distance along with the model training.
- In addition, convolutional filters that are the main component of our models, along with point mutations, capture more *complicated mutation patterns*, for example, the patterns taking into account the amino acid neighbor effect [11,63].

Results of the temporal experiments presented in Section 3.1 suggest that, among all proposed networks, the M23 network achieves MAE of about 0.935 antigenic units on average. As was established by the WHO Global Influenza Surveillance and Response System (GISRS), the variation of titer of less than one antigenic unit between two viruses is considered negligible [62]. This indicates that the M23 network has the potential to be used in the prediction of antigenic evolution based on the genetic sequence.

Among others, our temporal experiments were aimed at answering the question of how small a prehistoric time frame could be to keep sufficient information and to predict antigenic evolution in a target year. As it follows from Table 3, the Top Five networks in experiments $T_{all}$ and $T_{5}$ gained MAE of less than one antigenic unit. This proves that even a 5-year prehistory guarantees high-precision prediction of the antigenic distances, which in turn can help to increase the reliability of the models and speed up their training. This finding coincides with the known fact that the influenza virus antigenically changes every 2–5 years, which forces the vaccine composition to be reconsidered [64].

Surprisingly, in the results of temporal experiments, a significant MAE peak is observed for the year 2007 (see Figure 9a–Figure 12a). As demonstrated by Harvey et al. Figure 2 in [62], the vaccine strains *A/Solomon Islands/3/2006 (H1N1) -like virus* and *A/Brisbane/59/2007 (H1N1)-like virus* are antigenically different, at least on 0.5 antigenic units. In our opinion, the observed peak may have been caused by a new mutation pattern emerging in the year 2007, which the network did not meet before.

The SqueezeNet, used as a baseline model in our experiments, is one of the most compact best-performing image classification convolutional neural networks. Although the size of our best counter-part model M23 was about 90K greater than for SqueezeNet, as it follows from Table 3, in our Top Five list of our experiments, there are much more compact models of a very close performance. For instance, for $T_{all}$ and $T_{5}$, the sizes of the networks M11 and M10 were about 8 and 36 times less than the size of SqueezeNet, respectively.

By ignoring the direction of evolution, we reproduced the experiment scenarios proposed in [18] to evaluate the capacity of convolutional neural networks for antigenic distance prediction in comparison with conventional approaches based on substitutions or construction of the phylogenetic tree. The result of Table 4 shows that the M23 network is able to predict antigenic distance with the average MAE 0.871 antigenic unit for any pair of viruses not included in the training dataset. This error is noticeably lower than corresponding errors of this model observed in temporal experiments $T_{all}$ and $T_{5}$. A possible reason for this difference could be the non-anticipating nature of these experiments.

In summary, to the best of our knowledge, this paper is the first time where a convolutional neural network was applied to predict antigenic distance. The obtained results indicate that our best-performing network, M23, has great potential in modeling antigenic evolution based on genomic sequences. Our results can be employed in the reconstruction of antigenic cartography for the influenza virus. Furthermore, deep feature maps of our trained CNN models can reveal some hidden antigenic characteristics of the emerging novel viruses in the upcoming season, which seems to be essential for seasonal vaccine selection. Application of the proposed approach is not restricted to the prediction of influenza virus antigenicity. We believe that our approach can also be applied to other prediction problems, where among them are protein family classification, drug-target interaction prediction, and protein secondary structure prediction.

## 5. Conclusions

The prediction and assessment of antigenic evolution is a relevant and active task in influenza vaccine production. The early prediction of the antigenic distance can inform us about emerging novel antigenic variants that are crucial for vaccine combination. In this paper, we present an antigenic distance approximation approach based on convolutional neural networks. Our approach provides a multi-representation of input data encoding the HA sequence by amino acid physicochemical properties. The proposed networks automatically perform feature engineering, while taking into account the amino acid neighbor effect. The optimized network, determined by Grid Search, achieves the mean absolute error of about 0.935 antigenic units for yearly prediction of the HI assay over the years 2001–2009. By performing the training using different prehistory time frames, we found out that even 5 years prehistory is sufficient to model and predict the antigenic evolution.

The proposed approach accepts input data represented in the form of HA sequences. For future work, we plan to incorporate information about tertiary protein structure, which will likely lead to a significant improvement in overall accuracy of CNN-based prediction models. Furthermore, the obtained genetic signatures will be analyzed to build a relation between high-impact positions in the protein and the antigenicity of the virus. In addition, we plan to investigate the applicability of more modern neural network architectures, including recurrent and generative adversarial neural networks and reinforcement learning techniques to increase the overall prediction accuracy of the antigenic evolution.

## Figures and Tables

**Table 1:** Antigenic and primary sialic acid receptor-binding sub-domains in HA1, taken from [53].

Epitope Name	Sub-Domain
antigenic site Ca	137, 138, 139, 140, 141, 142, 166, 167, 168, 169, 170, 203, 204, 205, 221, 222, 235, 236, 237
antigenic site Cb	69, 70, 71, 72, 73, 74
antigenic site Sa	124, 125, 153, 154, 155, 156, 157, 159, 160, 161, 162, 163, 164
antigenic site Sb	184, 185, 186, 187, 188, 189, 190, 191, 192, 193, 194, 195
receptor-binding site	94, 131, 133, 150, 152, 180, 187, 191, 223, 225

**Table 2:** Hyperparameters of the examined networks M1–M32.

Model Name	Number of Convolution Layers	Number of Kernels	Kernel Size	Total Number of Parameters (K = 1000, M = 1,000,000)
M1	1	32	(7×2)	109 K
M2	1	32	(11×2)	72 K
M3	1	64	(7×2)	428 K
M4	1	64	(11×2)	274 K
M5	1	128	(7×2)	1.7 M
M6	1	128	(11×2)	1.1 M
M7	1	256	(7×2)	6.7 M
M8	1	256	(11×2)	4.2 M
M9	2	32	(7×2), (5×1)	29 K
M10	2	32	(11×2), (7×1)	24 K
M11	2	64	(7×2), (5×1)	104 K
M12	2	64	(11×2), (7×1)	81 K
M13	2	128	(7×2), (5×1)	397 K
M14	2	128	(11×2), (7×1)	293 K
M15	2	256	(7×2), (5×1)	1.5 M
M16	2	256	(11×2), (7×1)	1.1 M
M17	3	32	(7×2), (5×1),(3×1)	19 K
M18	3	32	(11×2), (7×1),(5×1)	23 K
M19	3	64	(7×2), (5×1),(3×1)	67 K
M20	3	64	(11×2), (7×1),(5×1)	77 K
M21	3	128	(7×2), (5×1),(3×1)	250 K
M22	3	128	(11×2), (7×1),(5×1)	277 K
M23	3	256	(7×2), (5×1),(3×1)	957 K
M24	3	256	(11×2), (7×1),(5×1)	1 M
M25	4	32	(7×2), (5×1), (3×1), (3×1)	19 K
M26	4	32	(11×2), (7×1), (5×1), (3×1)	26 K
M27	4	64	(7×2), (5×1), (3×1), (3×1)	67 K
M28	4	64	(11×2), (7×1), (5×1), (3×1)	89 K
M29	4	128	(7×2), (5×1), (3×1), (3×1)	249 K
M30	4	128	(11×2), (7×1), (5×1), (3×1)	326 K
M31	4	256	(7×2), (5×1), (3×1), (3×1)	957 K
M32	4	256	(11×2), (7×1), (5×1), (3×1)	1.2 M

**Table 3:** Top Five models for the temporal experiments. Average MAE <1 are highlighted.

Model Name	2001	2002	2003	2004	2005	2006	2007	2008	2009	Mean	STD
Experiment $T_{all}$: full prehistory											
M23	0.965	0.719	0.939	0.900	0.673	0.943	1.224	1.020	1.280	**0.935**	0.165
M11	1.030	0.713	0.961	0.910	0.633	0.900	1.299	1.013	1.097	**0.951**	0.198
M15	0.947	0.818	0.999	0.950	0.655	0.967	1.122	1.068	1.070	**0.955**	0.143
M13	1.027	0.825	0.971	0.948	0.615	0.935	1.241	1.034	1.065	**0.962**	0.172
M21	0.945	0.870	1.046	0.876	0.645	1.084	1.187	1.079	0.994	**0.970**	0.159
SqN	1.007	0.985	0.955	0.978	0.967	1.135	1.413	1.140	1.141	1.080	0.139
Experiment $T_{5}$: five years											
M23	0.890	0.836	0.964	0.924	0.657	1.039	1.186	1.092	1.134	**0.970**	0.165
M13	1.032	0.887	1.063	0.919	0.656	0.941	1.135	1.074	1.080	**0.976**	0.146
M10	1.004	1.034	1.008	0.962	0.528	0.943	1.197	1.119	1.005	**0.978**	0.186
M15	0.962	0.975	1.034	0.911	0.633	0.990	1.170	1.082	1.127	**0.987**	0.157
SqN	0.870	0.830	0.994	0.985	0.590	1.048	1.447	1.061	1.128	**0.995**	0.220
Experiment $T_{4}$: four years											
SqN	0.877	1.110	0.949	0.887	0.618	0.882	1.330	1.133	1.038	**0.981**	0.191
M24	0.992	0.798	0.975	0.921	0.893	1.054	1.519	1.077	1.129	1.040	0.206
M10	0.992	0.986	1.102	0.926	0.644	1.090	1.556	1.066	1.025	1.043	0.237
M16	1.029	0.780	1.067	0.933	0.871	1.054	1.465	1.105	1.147	1.050	0.195
M15	1.003	0.834	0.957	0.944	0.771	0.954	1.796	1.090	1.143	1.055	0.301
Experiment $T_{3}$: three years											
SqN	1.008	0.961	0.982	0.978	0.631	1.166	1.549	1.251	1.084	1.068	0.235
M16	0.945	0.748	0.985	0.934	0.850	1.281	1.618	1.149	1.125	1.070	0.262
M24	0.950	0.830	0.959	0.942	1.044	1.301	1.476	1.067	1.072	1.071	0.200
M23	0.924	0.807	0.928	0.945	0.869	1.290	1.671	1.114	1.166	1.079	0.271
M9	1.015	0.813	0.102	0.959	0.643	0.319	1.788	1.080	1.025	1.085	0.323

**Table 4:** Cross-validation results in terms of averaged Mean Absolute Error.

Model Name	Average MAE	STD
Titer		
M23	0.58	0.020
SqN	0.627	0.024
Virus		
M23	0.871	0.154
SqN	0.895	0.154

## Abbreviations

The following abbreviations are used in this manuscript:

CNN	Convolutional neural network
FE	Feature engineering
GISAID	Global Initiative on Sharing All Influenza Data
GISRS	Global Influenza Surveillance and Response System
HA	Hemagglutinin
HI	Hemagglutination inhibition
MAE	Mean absolute error
NA	Neuraminidase
PCA	Principal component analysis
WHO	World Health Organization
